# Rescue of glucocorticoid-programmed adipocyte inflammation by omega-3 fatty acid supplementation in the rat

**DOI:** 10.1186/1477-7827-12-39

**Published:** 2014-05-13

**Authors:** Peter J Mark, Caitlin S Wyrwoll, Intan S Zulkafli, Trevor A Mori, Brendan J Waddell

**Affiliations:** 1 School of Anatomy, Physiology & Human Biology, The University of Western Australia, Perth, Australia; 2 School of Medicine and Pharmacology, The University of Western Australia, Perth, Australia

## Abstract

**Background:**

Adverse fetal environments predispose offspring to pathologies associated with the metabolic syndrome. Previously we demonstrated that adult offspring of dexamethasone-treated mothers had elevated plasma insulin and pro-inflammatory cytokines, effects prevented by a postnatal diet enriched with omega (n)-3 fatty acids. Here we tested whether prenatal glucocorticoid excess also programmed the adipose tissue phenotype, and whether this outcome is rescued by dietary n-3 fatty acids.

**Methods:**

Offspring of control and dexamethasone-treated mothers (0.75 μg/ml in drinking water, day 13 to term) were cross-fostered to mothers on a standard (Std) or high n-3 (Hn3) diet at birth. Offspring remained on these diets post-weaning, and serum and retroperitoneal fat were obtained at 6 months of age (n = 5-8 per group). Serum was analysed for blood lipids and fatty acid profiles, adipocyte cross sectional area was measured by unbiased stereological analysis and adipose expression of markers of inflammation, glucocorticoid sensitivity and lipid metabolism were determined by RT-qPCR analysis.

**Results:**

Serum total fatty acid levels were elevated (*P* < 0.01) in male offspring of dexamethasone-treated mothers, an effect prevented by Hn3 consumption. Prenatal dexamethasone also programmed increased adipose expression of *Il6*, *Il1b* (both *P* < 0.05) and *Tnfa* (*P* < 0.001) mRNAs regardless of fetal sex, but again this effect was prevented (for *Il6* and *Il1b*) by Hn3 consumption. Offspring of dexamethasone-treated mothers had increased adipose expression of *Gr* (*P* = 0.008) and *Ppara* (*P* < 0.05) regardless of sex or postnatal diet, while *11bHsd1* was upregulated in males only. The Hn3 diet increased *Ppard* expression and reduced adipocyte size in all offspring (both *P* < 0.05) irrespective of prenatal treatment.

**Conclusions:**

Prenatal glucocorticoid exposure programmed increased expression of inflammatory markers and enhanced glucocorticoid sensitivity of adipose tissue. Partial prevention of this phenotype by high n-3 consumption indicates that postnatal dietary manipulations can limit adverse fetal programming effects on adipose tissue.

## Background

The incidence of obesity and related metabolic disorders (hypertension, insulin resistance and dyslipidemia) have risen dramatically in recent decades, with major implications for cardiovascular disease risk [1]. There is growing evidence that a range of insults in early life, including in utero, can developmentally program adverse outcomes for the adult phenotype. Most notably, undernutrition, overnutrition and glucocorticoid excess can all program adverse phenotypic outcomes. Accordingly, offspring born small (a proxy for a poor fetal environment) are predisposed to several adult-onset diseases including diabetes and obesity [2,3]. We previously demonstrated that offspring of rat mothers treated with dexamethasone during pregnancy were growth retarded at birth and as adults, were hyperleptinemic, hyperinsulinemic and hypertensive [4]. These offspring also exhibited a pro-inflammatory state, with plasma levels of tumor necrosis factor alpha (TNFα), interleukin 6 (IL6) and IL1β all increased by prenatal dexamethasone [5]. Moreover, because this pro-inflammatory state and most other adverse programming outcomes were prevented by postnatal dietary omega (n)-3 fatty acid supplementation, we have proposed that inflammation may be a key driver of the overall programmed phenotype [5]. In particular, adipose tissue inflammation may underlie the programmed pro-inflammatory phenotype, since adipose tissue leptin expression was elevated in offspring that had been exposed prenatally to dexamethasone [4]. To test this hypothesis we characterised the adipose tissue phenotype and serum fatty acid profiles in adult offspring of dexamethasone-treated mothers. Specifically, we measured adipocyte size (by unbiased stereology) and adipose expression of pro-inflammatory cytokines, peroxisome proliferator-activated receptors, and determinants of glucocorticoid sensitivity (i.e., the glucocorticoid receptor (GR; *Nr3c1*) and 11β-hydroxysteroid dehydrogenase (*11bHsd*) enzymes). This focus on adipose glucocorticoid sensitivity was considered important given that enhanced glucocorticoid activity in adipocytes appears to be obesogenic (for review see [6]). Moreover, because the programmed phenotype in our model was largely rescued in offspring raised on a diet enriched with n-3 fatty acids [4,5] we also characterised the adipose phenotype and fatty acid profiles of these offspring.

## Methods

### Animals and diets

Nulliparous albino Wistar rats aged between 8 and 10 weeks were obtained from the Animal Resources Centre (Murdoch, Australia) and maintained under controlled lighting and temperature as previously described [7]. Two isocaloric, semi-pure diets were used in this study, each formulated with identical ratios of protein, carbohydrate, fat, and salt, but with markedly different n-3 fatty acid contents as previously described [4]. The Hn3 diet contained 34% of total fats as long chain n-3 fatty acids whereas the standard diet contained 0.8% n-3 fatty acids. The semi-pure diets were manufactured by Specialty Feeds (Glen Forrest, Australia) and were sterilized by gamma-irradiation. Ten days before mating, half the females were placed on one of the two semi-pure diets; standard (Std) or high omega-3 fatty acids (Hn3), while the others remained on a normal rat chow (Specialty Feeds, Glen Forrest, Australia). All rats were provided with acidified water and food *ad libitum*. All procedures involving animals were approved by the Animal Ethics Committee of The University of Western Australia. Rats were mated overnight and the day on which spermatozoa were present in a vaginal smear was designated day 1 of pregnancy. Dexamethasone (Dex) acetate (Sigma Chemical Co., St Louis, MO, USA) was administered in the drinking water (0.75 μg/ml) from day 13 until birth in half of the pregnancies. Previous studies show that dexamethasone acetate administered via maternal drinking water results in consistent, dose-dependent reductions in birth weight [8]. In the current study, birth weight was reduced by 24% and 25% in males and females, respectively, as previously reported [4]. Within 24 h of birth, all pups from control (Con) and Dex-treated mothers were cross-fostered to a mother consuming either a Std diet or Hn3 diet. Cross-fostering resulted in four treatment groups (Con/Std, Con/Hn3, Dex/Std and Dex/Hn3), and pups remained with their foster mothers until weaning, at which point male and female offspring were caged separately and remained on their allocated diets (Std or Hn3).

### Tissue collection

At 6 months of age, a male and female were randomly selected from each litter, fasted overnight for 16 h, weighed and anesthetized with 40 mg/kg Nembutal (Rhone Merieux, Pinkenba, Australia). Blood was collected from the dorsal aorta and serum stored at −20°C until subsequent analysis. Retroperitoneal adipose tissue samples were collected and either fixed (Histochoice) or snap frozen in liquid nitrogen and stored at −80°C until analysis.

### Measurement of blood lipids

Serum total cholesterol and triacylglycerols were determined enzymatically on a Cobas MIRA analyser (Roche Diagnostics, Basel, Switzerland) with reagents from Trace Scientific (Melbourne, Australia). High density lipoprotein-cholesterol (HDL-C) was determined on a heparin-manganese supernatant [9]. The intra-assay coefficients of variation were 2.2% at 4.2 mM and 1.4% at 10.5 mM for total cholesterol, 1.6% at 4.0 mM and 2.5% at 1.2 mM for triacylglycerols, and 1.9% at 1.1 mM for HDL-C. In each case all samples were measured in a single assay. Serum levels of a range of fatty acids were analysed by gas chromatography as previously described [10]. Briefly, serum (200 μL) was extracted with 2 ml chloroform/methanol (2:1, v/v). Fatty acid methyl esters were prepared by treatment of total lipid extracts with 4% H_2_SO_4_ in methanol at 90°C for 20 min and analysed by gas liquid chromatography using a Hewlett-Packard model 5980A gas chromatograph (Hewlett Packard, Rockville, MD). The samples were resolved on a BPX70 column (25 cm × 0.32 mm, 0.25 μm film thickness; SGE, Ringwood, Victoria, Australia) with a temperature program increasing from 150°C to 210°C at 4°C/min and using N_2_ as the carrier gas at a split ratio of 30:1. Peaks were identified by comparison with a known standard mixture.

### Adipose tissue morphometry

Fixed retroperitoneal fat samples (n = 5 per group) from male rats were processed and embedded in paraffin wax. Three sections (3 μm thick) were cut (random starting section and orientation) with a distance between sections of 300 μm. Sections were stained with hematoxylin and eosin before dehydration in graded alcohols and mounting in DPX for morphometrical analysis. Estimation of the mean area of unilocular adipocytes was performed using an unbiased stereological technique which involved a Cavalieri grid overlay (grid size: 20 μm) [11]. Images were observed at x 20 magnification and 100 cells per section in 3 sections were chosen by systematic uniform sampling. Grid points falling onto the region of interest were counted (Stereo Investigator, MBF Bioscience, Williston, VT) and area estimates were calculated by multiplying the number of grid points counted by the area associated per grid point [12]. Results were adjusted for shrinkage during processing as determined by the measurement of 100 erythrocyte diameters and comparison with the standard diameter of erythrocytes in Wistar rats [13].

### Measurement of mRNA expression by quantitative RT-PCR analysis

Total RNA was extracted from tissue samples using an RNeasy Lipid Tissue kit (Qiagen, Melbourne, Australia). RNA (1 μg) was reverse transcribed at 42°C for 120 min using murine Moloney Leukemia Virus Reverse Transcriptase (Promega, Madison, WI, USA) according to the manufacturer’s instructions and supplemented with 2.5 mg/mL Ficoll 400 and 7.5 mg/mL Ficoll 70 [14]. The resultant cDNA was purified using the UltraClean PCR Clean-up Kit (MoBio Laboratories Inc., Solana Beach, CA, USA). All primers, other than *Il6, Il1b* and *Rpl19*, were designed using Primer3 software [15], and were positioned to span introns to prevent amplification from contaminating genomic DNA; ribosomal *Rpl19* was used as an internal control [16]. Quantitect primer sets for *Il6* (QT00182896) and *Il1b* (QT00181657) were obtained from Qiagen (Melbourne, Australia) and amplified in Quantifast SYBR Green PCR mix according to the manufacturer’s instructions. For each of the remaining genes, the PCR primer sequences are shown in the Table 1 along with the presence or absence of 2.5 mg/mL Ficoll 400 and 7.5 mg/mL Ficoll 70 [14], MgCl_2_ concentrations, annealing temperatures, and PCR product sizes. External standards were generated from regular PCR products and ten-fold serial dilutions of the PCR product were made in RNase-free water (1- to 10^7^ - fold dilutions). Quantitative PCR was performed in 10 μL reaction volumes using the Rotor-Gene 6000 system (Corbett Research, Sydney, Australia) with primer concentrations as specified in the Table 1, Immolase enzyme (0.5 U; Bioline, Alexandria, Australia), and 1/40 000 dilution of stock SYBR Green (Molecular Probes, Eugene, OR, USA) per reaction. The PCR cycling conditions included an initial denaturation at 94°C for 10 min followed by 45 cycles at 94°C for 1 s; an annealing temperature (specified in Table 1) for 15 s; and 72°C for 5 s. In each case, melt-curve analysis from 70 to 99°C showed a single PCR product that was confirmed to be the correct size and sequence by gel electrophoresis and sequence analysis respectively (data not shown). Fluorescence values were analyzed, standard curves constructed using the RotorGene software (Corbett Research, Sydney, Australia), and all samples standardized against a reference control (*Rpl19*).

**Table 1:** Primer sequences and conditions for quantitative PCR

***Gene***	***Sequence***	***Cycling conditions (45 cycles)***	***Ficoll***	***MgCl***_***2 ***_ ***(mM)***	**Product size (bp)**
*11bHsd1*	F: 5` ctctctgtgtcctcggcttc 3`	95°C/1 sec	No	2.5	131
	R: 5` ttccatgatcctccttcctg 3`	57°C/15 sec			
		72°C/5 sec			
*Adiponectin*	F: 5` tggcagagatggcactcc 3`	95°C/1 sec	No	4	101
	R: 5` cttccgctcctgtcattcc 3`	59°C/15 sec			
		72°C/5 sec			
*GR*	F: 5` cttgagaaacttacacctcgatgacc 3`	95°C/1 sec	Yes	4.5	461
	R: 5` agcagtaggtaaggagattctcaacc 3`	62°C/20 sec			
		72°C/30 sec			
*Il1b*	QT00182896	95°C/1 sec	No	-	-
		60°C/30 sec			
*Il6*	QT00181657	95°C/1 sec	No	-	-
		60°C/30 sec			
*Pgc1a*	F: 5` tctggaactgcaggcctaactc 3`	95°C/1 sec	No	4	96
	R: 5` gcaagagggcttcagctttg 3`	60°C/15 sec			
		72°C/5 sec			
*Ppara*	F: 5` aatccacgaagcctacctga 3`	95°C/1 sec	Yes	2.5	132
	R: 5` gtcttctcagccatgcacaa 3`	60°C/15 sec			
		72°C/5 sec			
*Ppard*	F: 5` gaggggtgcaagggcttctt 3`	95°C/1 sec	No	2.5	101
	R: 5` cacttgttgcggttcttctg 3`	60°C/15 sec			
		72°C/5 sec			
*Pparg*	F: 5` catgcttgtgaaggatgcaag 3`	95°C/1 sec	No	3	131
	R: 5` ttctgaaaccgacagtactgacat 3`	63°C/15 sec			
		72°C/5 sec			
*Tnfa*	F: 5` tactgaacttcggggtgattggtcc 3`	95°C/1 sec	No	2	295
	R: 5` cagccttgtcccttgaagagaacc 3`	60°C/30 sec			
*Rpl19*	F: 5`ctgaaggtcaaagggaatgtg 3`	95°C/1 sec	Yes	3	195
	R: 5`ggacagagtcttgatgatctc 3`	52°C/15 sec			
		72°C/5 sec			

### Statistical analysis

All data are expressed as mean ± S.E.M., with each litter representing an ‘*n*’ of one. Analysis of variance (ANOVA) was used to attribute variation to sex, prenatal treatment, and postnatal diet. Where the *F* test was statistically significant (*P* < 0.05), specific differences were assessed by *post hoc* least significant difference (LSD) tests [17]. When a significant interaction term was observed, further analyses of data subsets were made by ANOVA or unpaired *t* tests as appropriate.

## Results

### Blood lipid profiles and adipocyte size

**Figure 1:**
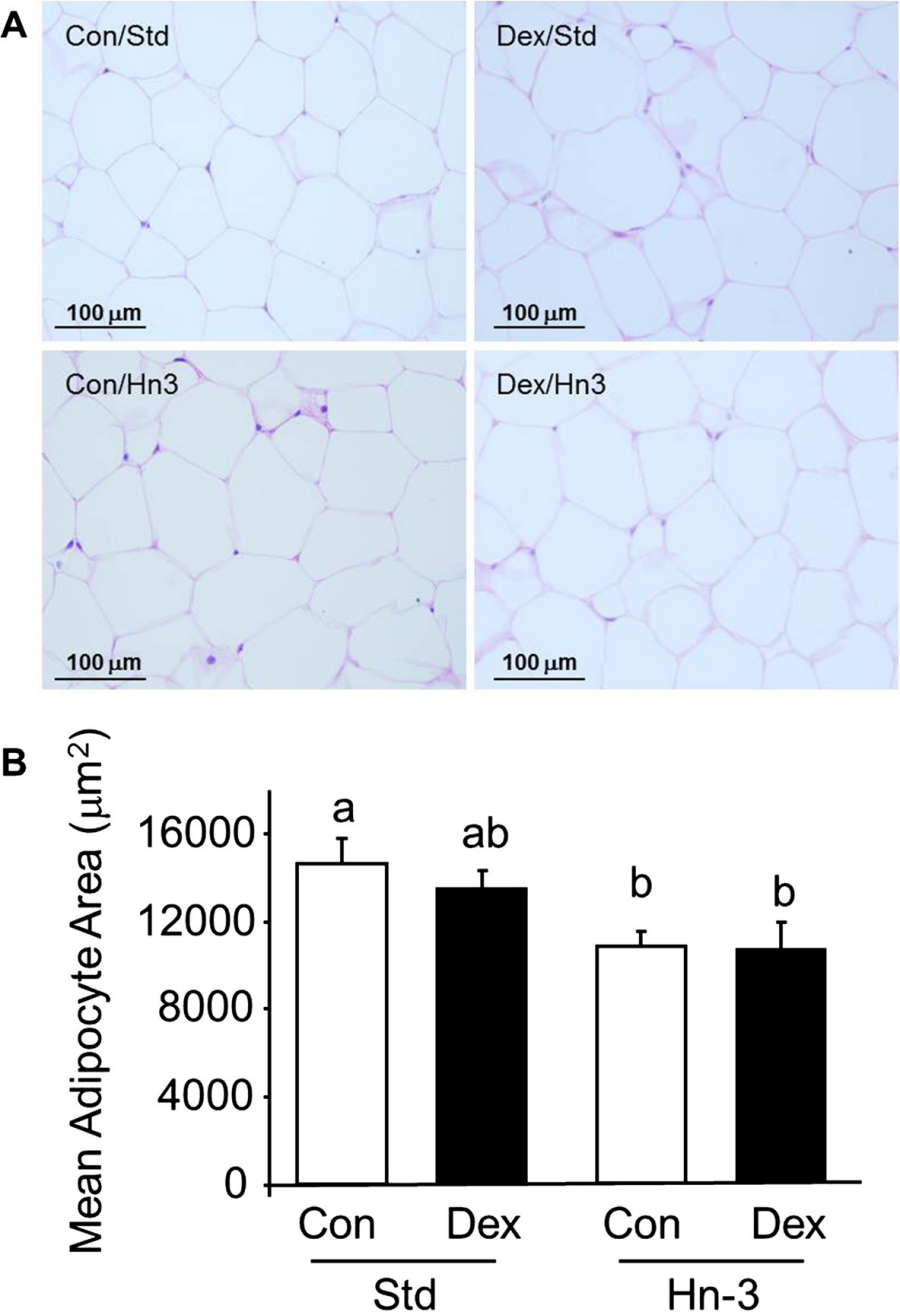
**Interactive effects of prenatal dexamethasone exposure and postnatal diet on adipose tissue cross-sectional area at 6 months of age in male rats.** Histological images of retroperitoneal adipose tissue **(A)** and stereological quantitation of cross-sectional area of adipocytes **(B)**. Control (Con) group in clear bars, Dex-exposed (Dex) group in black bars. Values are mean ± SEM (n = 5-8). Values without common notation differ significantly (*P* < 0.05; one-way ANOVA, LSD).

As previously reported male and female offspring of dexamethasone-treated mothers in this cohort were born smaller and had not shown catch-up growth by 6 months of age [4]. Male and female offspring that consumed the Std diet had comparable levels of serum triacylglycerols, cholesterol and HDL-C (Table 2). Postnatal consumption of the Hn3 diet lowered (*P* < 0.05) serum triacylglycerols (by 40-50% in males; 30-55% in females) and cholesterol (by 25-30% in males; 15-25% in females). Consumption of the Hn3 diet also reduced (by 20%; *P* = 0.02) adipocyte cross sectional area in male offspring irrespective of prenatal treatment (Figure 1B).

**Table 2:** Serum triacylglycerols, cholesterol and HDL-C in 6-month old offspring of control (Con) and dexamethasone (Dex)-treated mothers

	**Con/Std**	**Dex/Std**	**Con/Hn3**	**Dex/Hn3**
**Triaclyglycerols (mM)**				
Male	1.05 ± 0.23	0.75 ± 0.05	0.51 ± 0.03*	0.44 ± 0.04*
Female	0.89 ± 0.12	1.16 ± 0.16	0.62 ± 0.06*	0.53 ± 0.05*
**Cholesterol (mM)**				
Male	2.53 ± 0.30	2.20 ± 0.11	1.94 ± 0.14*	1.53 ± 0.14*
Female	2.47 ± 0.16	2.05 ± 0.09	1.88 ± 0.07*	1.73 ± 0.19*
***HDL-C (mM)***				
Male	1.93 ± 0.30	1.75 ± 0.11	1.57 ± 0.14	1.29 ± 0.14
Female	1.67 ± 0.16	1.34 ± 0.10	1.41 ± 0.07	1.39 ± 0.19

Offspring were raised on either a standard (Std) or a high omega-3 (Hn3) diet.

Values are the mean + SEM (*n* = 5–8 per group).

*P < 0.05 compared to corresponding Std diet (three-way ANOVA and LSD test).

### Serum fatty acid profile

Total fatty acid levels in serum were substantially elevated (81%; P < 0.01) in male (Table 3) but not female (data not shown) offspring of dexamethasone-treated mothers raised on the Std diet. In contrast, prenatal dexamethasone had minimal effect on serum fatty acid levels of offspring raised on the Hn3 diet (Table 2) with only C16:0, C18:0 and total fatty acid content increased. As expected, however, offspring raised on the Hn3 diet had elevated levels of all n-3 fatty acids, but lower levels of the n-6 fatty acids C20:4 and C22:4. Dietary effects in female offspring broadly paralleled those in males (results not shown).

**Table 3:** Serum concentrations of fatty acids (μg/mL) in male offspring at 6 months of age

	**Con/Std**	**Dex/Std**	**Con/Hn3**	**Dex/Hn3**
***C14:0***	8 ± 1^a^	20 ± 2^b^	11 ± 1^ac^	16 ± 3^bc^
***C16:0***	155 ± 7^a^	289 ± 19^b^	165 ± 8^a^	215 ± 18^c^
***C18:0***	83 ± 9^a^	140 ± 9^b^	46 ± 5^c^	63 ± 9^a^
***C16:1(n-7)***	33 ± 4^a^	65 ± 9 ^b^	53 ± 7 ^b^	64 ± 3 ^b^
***C18:1 (n-9)***	175 ± 15^a^	315 ± 28^b^	94 ± 8^c^	120 ± 11^c^
***C18:2 (n-6)***	53 ± 6^a^	125 ± 11^b^	58 ± 3^a^	79 ± 11^a^
***C20:3 (n-6)***	5 ± 1^a^	8 ± 1^b^	3 ± 0.5^a^	4 ± 0.5^a^
***C20:4 (n-6)***	225 ± 46^a^	372 ± 41^b^	63 ± 4^c^	73 ± 5^c^
***C20:5 (n-3)***	2 ± 0.5^a^	4.6 ± 0.4^b^	97 ± 9^c^	108 ± 14^c^
***C22:4 (n-6)***	3 ± 0.5^a^	9 ± 1^a^	0.5 ± 0.5^b^	0.5 ± 0.5^b^
***C22:5 (n-3)***	3 ± 0.5^a^	6 ± 1^b^	16 ± 1^c^	22 ± 3^c^
***C22:6 (n-3)***	25 ± 2^a^	46 ± 5^b^	66 ± 4^c^	83 ± 13^c^
***Total n6***	228 ± 46^a^	354 ± 48^b^	63 ± 4^c^	74 ± 5^c^
***Total n3***	30 ± 2^a^	54 ± 7^b^	179 ± 11^c^	213 ± 29^c^
***Total***	769 ± 65^ac^	1398 ± 86^b^	672 ± 27^a^	846 ± 94^c^

Data were analysed by two-way ANOVA followed by *post hoc* LSD tests.

Where there was an interaction between treatment and diet, separate t-tests were performed. For each fatty acid (i.e. within each row), values without common notation (a, b, c) differ significantly (*P* < 0.05).

Values are the mean ± SEM (*n* = 7 to 8 per group).

### Programming of adipose cytokine expression

**Figure 2:**
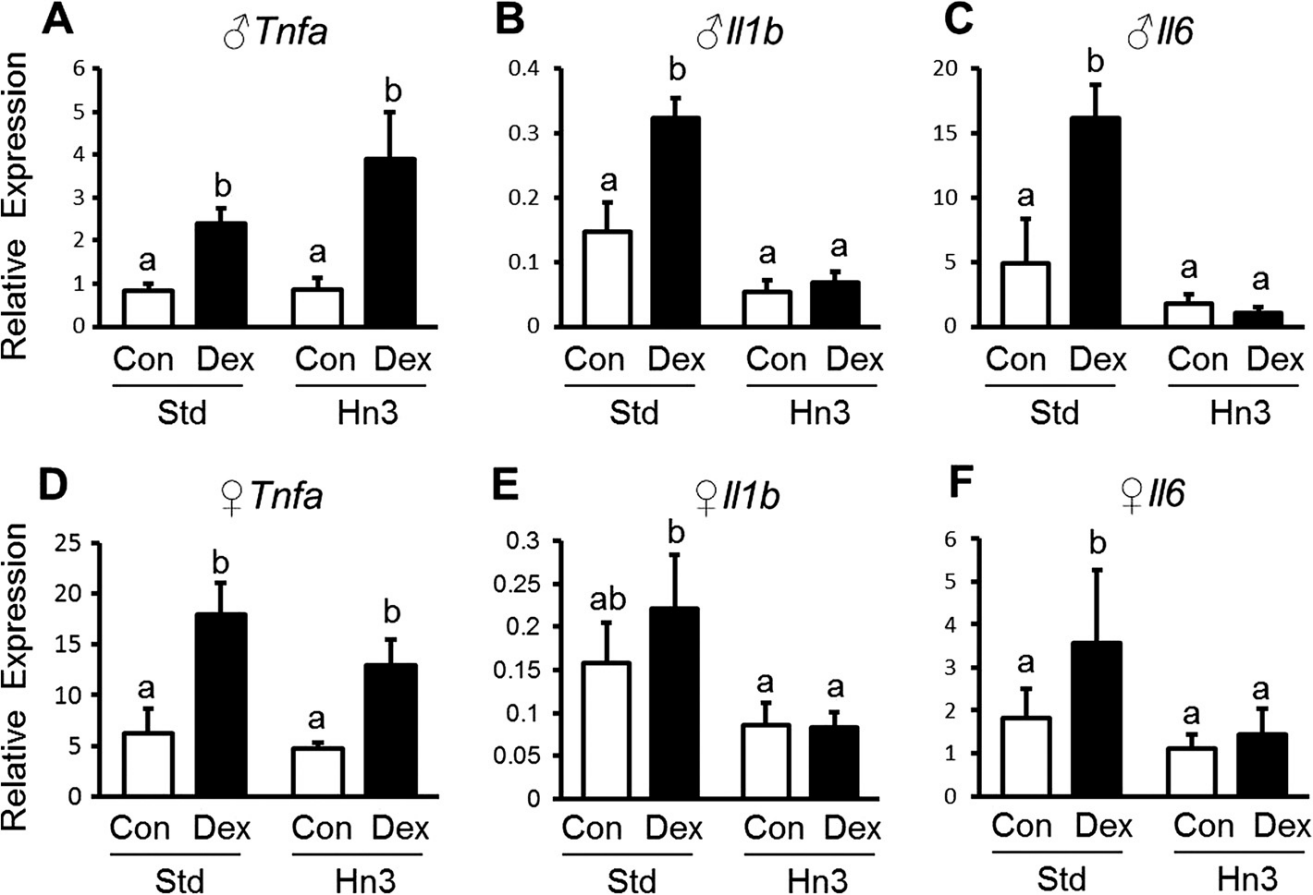
**Interactive effects of prenatal dexamethasone exposure and postnatal diet on *Tnfa *(A, D), *Il6 *(B, E) and *Il1b *(C, F) mRNA expression in adipose tissue in male (A-C) and female (D-F) rats.** Control (Con) group in clear bars, Dex-exposed (Dex) group in black bars. Values are mean ± SEM (n = 5-8). Values without common notation differ significantly (*P* < 0.05; two-way ANOVA, LSD).

Adipose expression of both *Il6* and *Il1b* mRNAs were upregulated by prenatal dexamethasone exposure (1.6-3-fold, *P* < 0.05) in male and female offspring raised on a Std diet, but these programmed increases were prevented in offspring raised on the Hn3 diet (Figure 2). Adipose expression of *Tnfa* mRNA was also elevated (2–3.5-fold, *P* < 0.001) in offspring of dexamethasone-treated mothers (Figure 2) regardless of postnatal diet. There was also a sex effect on adipose *Tnfa* expression (males < females; *P* = 0.006), and adipose expression of adiponectin was not affected by either prenatal dexamethasone or postnatal diet (data not shown).

### Glucocorticoid sensitivity of adipose tissue

**Figure 3:**
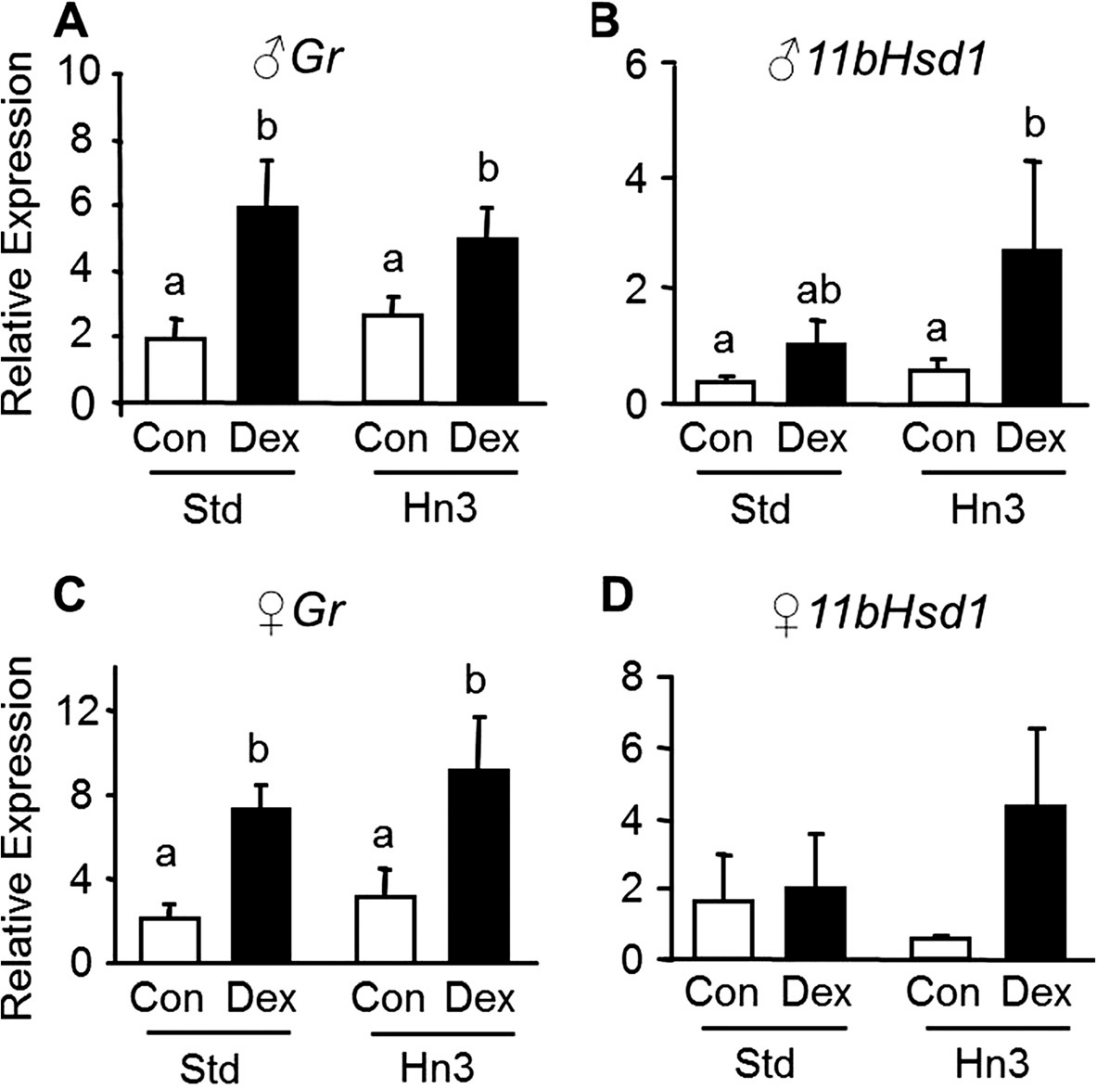
**Interactive effects of prenatal dexamethasone exposure and postnatal diet on *GR *(A, C) and *11bHsd1 *(B, D) mRNAs in adipose tissue in male (A-B) and female (C-D) rats.** Control (Con) group in clear bars; dexamethasone (Dex) group in black bars. Values are mean ± SEM (n = 5-8). Values without common notation differ significantly (*P* < 0.05; two-way ANOVA, LSD).

Maternal dexamethasone treatment increased (*P* < 0.05) adipose *GR* mRNA expression irrespective of diet in both male and female offspring (Figure 3A & C). Adipose expression of *11bHsd1* was also upregulated (*P* < 0.05) in male but not female offspring of dexamethasone-treated mothers, particularly those raised on the Hn3 diet (Figure 3B & D).

### Adipose expression of PPARs

**Figure 4:**
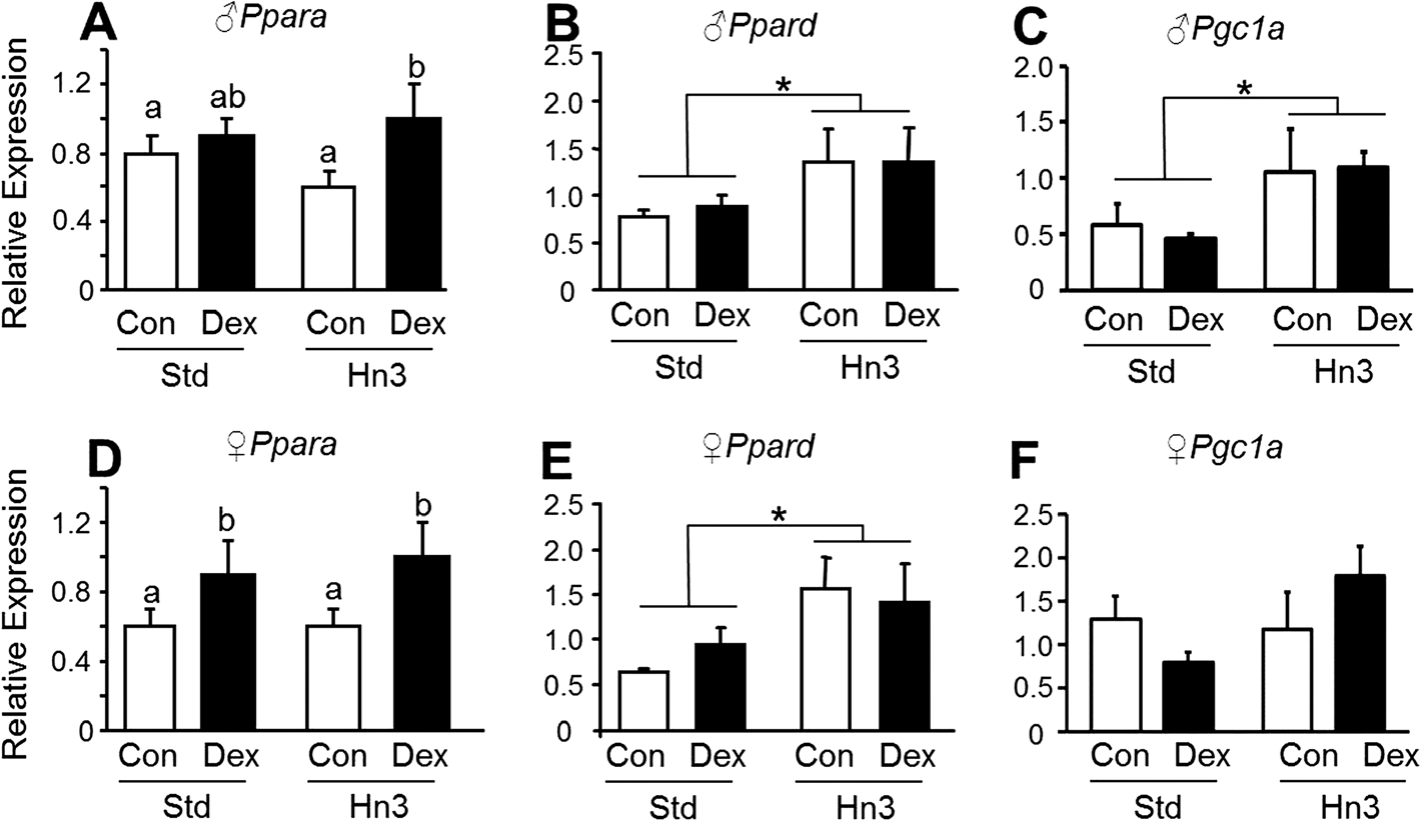
**Interactive effects of prenatal dexamethasone exposure and postnatal diet on *Ppara *(A, D), *Ppard *(B, E) and *Pgc1a *(C, F) mRNA expression in adipose tissue in male (A-C) and female (D-F) rats.** Control (Con) group in clear bars, Dex-exposed (Dex) group in black bars. Values are mean ± SEM (n = 5-8). Values without common notation differ significantly (*P* < 0.05; two-way ANOVA, LSD). *P < 0.05 compared to Std diet.

Maternal dexamethasone treatment increased adipose expression of *Ppara* (*P* = 0.008) in both male and female offspring regardless of diet (Figure 4). In contrast, *Ppard* expression was unaffected by prenatal dexamethasone but was upregulated (1.5-1.8-fold, *P* < 0.05) by consumption of the Hn3 diet at (Figure 4). Adipose expression of *Pparg* was unaffected by prenatal dexamethasone or postnatal diet in both males and females (results not shown). The PPAR co-activator, *Pgc1a*, was unaffected by prenatal treatment but was higher (*P* < 0.05) in male (but not female) offspring raised on the Hn3 diet (Figure 4C).

## Discussion

Fetal glucocorticoid excess is recognised as a key mechanism involved in the programming of adverse phenotypic outcomes in adult offspring, including hypertension and insulin resistance. The present study showed that prenatal dexamethasone exposure programmed increased adipose inflammation and serum fatty acid levels at 6 months of age, and that consumption of a postnatal diet enriched with n-3 fatty acids alleviated many of these adverse outcomes. Specifically, adipose expression of *Tnfa*, *Il6*, *Il1b*, *GR*, *11bHsd1* and *Ppara* and serum fatty acid levels (males only) were all elevated in adult offspring of dexamethasone-treated mothers. Consumption of a diet enriched with n-3 fatty acids from birth corrected the programmed increases in serum fatty acids and adipose expression of *Il6* and *Il1b*. Dietary supplementation with n-3 fatty acids also upregulated expression of *Ppard* and reduced mean adipocyte size regardless of prenatal treatment.

Disturbances to the normal fetal environment, such as excess glucocorticoid exposure or undernutrition, have been associated with a predisposition for adverse physiological outcomes in adult offspring including type II diabetes, hypertension and obesity in humans [18,19] and rats [4,5,20-22]. Our developmental programming model involves fetal glucocorticoid excess over the final third of rat pregnancy, which leads to fetal growth restriction [4,7,23] and subsequent development of offspring hypertension, hyperleptinemia [4], hyperinsulinemia and elevated plasma cytokine levels [5]. Although percent adiposity appeared unaffected in these programmed offspring [4], the present study shows that fetal glucocorticoid excess programmed marked changes in the adipose tissue phenotype. Most notably, adipose mRNA expression of the pro-inflammatory cytokines *Tnfa*, *Il6* and *Il1b* was elevated in male and female offspring of dexamethasone-treated mothers, consistent with our previous report showing that plasma levels of these cytokines were elevated in this same cohort of animals [5]. Furthermore, although insulin sensitivity was not measured in these animals, analysis of fasting insulin levels indicated that prenatal dexamethasone treatment resulted in hyperinsulinemia [5]. This suggests that the pro-inflammatory state of adipose tissue may contribute to systemic inflammation and insulin resistance. Moreover, programming of the pro-inflammatory adipose phenotype was largely prevented when offspring were raised on the Hn3 diet, which normalised both *Il6* and *Il1b* expression (but interestingly not that of *Tnfa*) and circulating insulin levels. This partial correction i.e. normalisation of *Il6* and *Il1b* but not *Tnfa*, parallels the effects of the Hn3 diet on systemic inflammatory state [5] and the anti-inflammatory effects of the high n-3 diet are consistent with observations in a range of previous animal and human studies (for review see [24]). Exactly why programmed *Tnfa* expression is not corrected is unclear and requires further investigation.

A second key feature of the programmed adipose tissue phenotype was an apparent increase in glucocorticoid sensitivity, a characteristic linked to the aetiology of obesity [25-27]. Thus, adipose expression of both the GR (*Nr3c1*) and *11bHsd1* mRNAs were both increased in offspring of dexamethasone-treated mothers. Glucocorticoid activation of the GR is enhanced by local expression of 11βHSD1, which catalyses regeneration of active corticosterone from inert 11-dehydrocorticosterone [28]. As such, these changes, coupled with increased stress-responsiveness of programmed offspring in this model [29], are likely to promote GR activation in adipose tissue. These observations extend those of Gnanalingham et al. (2005), who showed that prenatal dexamethasone exposure in a sheep model increased GR and 11βHSD1 levels in perirenal adipose tissue of newborns [30]. Our data are also consistent with the programmed upregulation of GR expression in kidney [31] and liver [32] following prenatal dexamethasone, and in adipose tissue after maternal undernutrition [22]. Collectively, these studies show that adipose glucocorticoid sensitivity is enhanced by fetal insults (nutritional or excess glucocorticoids).

This outcome might be expected to reduce adipose inflammation, given that glucocorticoids are normally potent anti-inflammatory agents. Indeed, glucocorticoids and pro-inflammatory cytokines are thought to act synergistically to stimulate *11bHsd1* expression in acute inflammation, thereby further enhancing local levels of active glucocorticoid to perhaps limit inflammation (for review see [6]). But if this acute inflammation is not resolved and chronic inflammation ensues, as in metabolic disease, the anti-inflammatory action of glucocorticoids appears to be lost [6]. Interestingly, a similar scenario is evident in the late gestation rat placenta, where increased expression of pro-inflammatory cytokines [33], *GR* and *11bHsd1*[23] all occur despite rising levels of maternal and fetal glucocorticoids.

The PPAR transcription factors play key roles in adipocyte differentiation and function, influencing the balance between fat storage (via PPARγ) and utilisation (via PPARδ) (for reviews see [34,35]). Although prenatal dexamethasone did not affect adipose expression of either *Pparg* or *Ppard*, it did program a marginal increase in adipose expression of *Ppara,* particularly in female offspring. This effect may have developed as a compensatory response to the heightened adipose inflammatory state, since PPARα activation is known to exert anti-inflammatory effects in other cell types [36]. Interestingly, these *Ppard* responses to diet are different to those observed in the skeletal muscle of the same rats [5], where elevated *Ppard* expression was programmed by prenatal Dex exposure, but was unaffected by diet. This likely represents tissue-specific regulation of *Ppard*.

Male offspring of dexamethasone-treated mothers showed elevated serum levels of total fatty acids, an effect evident across all fatty acid groups. While the specific reasons why this effect was limited to male offspring are not known, gender differences have been noted in fatty acid oxidation rates in humans (females lower than males; for review see [37]). Although previous studies have reported a programmed increase in serum free fatty acid and triacylglycerol levels (by maternal obesity; [38]), importantly this was observed in offspring that were themselves obese. In contrast, the programmed increase in serum fatty acids in the present study occurred in the absence of increased adiposity, possibly indicative of a greater susceptibility to obesogenic stimuli. It is possible that the programming of elevated serum fatty acids by dexamethasone may be linked, in part, to the elevated levels of pro-inflammatory cytokines [5], since these cytokines are known to increase lipolysis in both humans and rats [39,40]. In this context it is noteworthy that adipose expression of *Il6* was also corrected by the Hn3 diet.

Consumption of the Hn3 diet markedly reduced serum levels of triacylglycerols and cholesterol, but did not affect HDL-C levels, consistent with known effects of n-3 fatty acids on lipid profiles in humans [41]. This dietary effect may reflect inhibition of hepatic triacylglycerol synthesis and stimulation of beta-oxidation by n-3 fatty acids [42], effects that are likely to have contributed to the decreased adipocyte size in rats fed the Hn3 diet. Elevated expression of *Ppard* was also observed in these smaller adipocytes, consistent with previous studies in rats that consumed n-3 fatty acids [43]. Activation of PPARδ in adipocytes leads to improved lipid profiles and reduced adiposity [44] and n-3 fatty acids are known ligands for PPARs [45]. Decreased adipocyte size has also been associated with increased insulin sensitivity, a reduced incidence of type II diabetes in rats [46] and humans [47] as well as reduced expression and release of adipocytokines [48,49], suggestive of a less detrimental phenotype associated with the smaller adipocytes. Furthermore, this reduced adipocyte size is consistent with the observed decline in epididymal fat pad weight previously reported for these animals [4].

## Conclusions

This study demonstrates that prenatal dexamethasone exposure programmed detrimental changes in the adipocyte phenotype including a pro-inflammatory state and increased glucocorticoid sensitivity. Consumption of a postnatal diet enriched with n-3 fatty acids attenuated pro-inflammatory adipocytokine responses, decreased adipose cross-sectional area and alleviated many of these adverse programming effects of fetal glucocorticoid excess.

## Abbreviations

11βHSD /11bHsd: 11β hydroxysteroid dehydrogenase; ANOVA: Analysis of variance; GR: Glucocorticoid receptor; HDL: High density lipoprotein; Hn3: High omega3; IL: Interleukin; LSD: Least significant difference; MgCl2: Magnesium chloride; PGC1α/Pgc1a: PPAR gamma co-activator 1 alpha; PPAR/Ppar: Peroxisome proliferator activated receptor; RNA: Ribonucleic acid; RNase-free: Ribonuclease-free; RT-PCR: Reverse transcription polymerase chain reaction; Std: Standard; TNFα/Tnfa: Tumour necrosis factor alpha.

## Competing interests

The authors declare that there is no competing of interest that could be perceived as prejudicing the impartiality of the research reported.

## Authors’ contributions

PJM, CSW, TAM and BJW designed research; CSW, PJM, and ISZ conducted research and analysed data; PJM and BJW wrote the paper and BJW had primary responsibility for final content. All authors read and approved the final manuscript.

## References

[B1] Birdsall KM Vyas S Khazaezadeh N Oteng-Ntim E Maternal obesity: a review of interventions Int J Clin Pract 2009 63 494 507 10.1111/j.1742-1241.2008.01910.x 19222635

[B2] Thompson NM Norman AM Donkin SS Shankar RR Vickers MH Miles JL Breier BH Prenatal and postnatal pathways to obesity: different underlying mechanisms, different metabolic outcomes Endocrinology 2007 148 2345 2354 10.1210/en.2006-1641 17272392

[B3] Vuguin P Raab E Liu B Barzilai N Simmons R Hepatic insulin resistance precedes the development of diabetes in a model of intrauterine growth retardation Diabetes 2004 53 2617 2622 10.2337/diabetes.53.10.2617 15448092

[B4] Wyrwoll CS Mark PJ Mori TA Puddey IB Waddell BJ Prevention of programmed hyperleptinemia and hypertension by postnatal dietary omega-3 fatty acids Endocrinology 2006 147 599 606 10.1210/en.2005-0748 16210371

[B5] Wyrwoll CS Mark PJ Mori TA Waddell BJ Developmental programming of adult hyperinsulinemia, increased proinflammatory cytokine production, and altered skeletal muscle expression of SLC2A4 (GLUT4) and uncoupling protein 3 J Endocrinol 2008 198 571 579 10.1677/JOE-08-0210 18591261

[B6] Chapman KE Coutinho AE Zhang Z Kipari T Savill JS Seckl JR Changing glucocorticoid action: 11beta-Hydroxysteroid dehydrogenase type 1 in acute and chronic inflammation J Steroid Biochem Mol Biol 2013 137 82 92 23435016 10.1016/j.jsbmb.2013.02.002 PMC3925798

[B7] Burton PJ Waddell BJ 11 beta-Hydroxysteroid dehydrogenase in the rat placenta: developmental changes and the effects of altered glucocorticoid exposure J Endocrinol 1994 143 505 513 10.1677/joe.0.1430505 7836896

[B8] Smith JT Waddell BJ Increased fetal glucocorticoid exposure delays puberty onset in postnatal life Endocrinology 2000 141 2422 2428 10.1210/endo.141.7.7541 10875242

[B9] Warnick GR Albers JJ A comprehensive evaluation of the heparin-manganese precipitation procedure for estimating high density lipoprotein cholesterol J Lipid Res 1978 19 65 76 202660

[B10] Woodman RJ Mori TA Burke V Puddey IB Watts GF Beilin LJ Effects of purified eicosapentaenoic and docosahexaenoic acids on glycemic control, blood pressure, and serum lipids in type 2 diabetic patients with treated hypertension Am J Clin Nutr 2002 76 1007 1015 12399272 10.1093/ajcn/76.5.1007

[B11] Gundersen HJ Jensen EB The efficiency of systematic sampling in stereology and its prediction J Microsc 1987 147 229 263 10.1111/j.1365-2818.1987.tb02837.x 3430576

[B12] Weibel ER Stereological Methods: Practical Methods for Biological Morphometry 1979 London - New York - Toronto: Academic Press

[B13] Kinosita K Jr Tsong TY Survival of sucrose-loaded erythrocytes in the circulation Nature 1978 272 258 260 10.1038/272258a0 628451

[B14] Lareu RR Harve KS Raghunath M Emulating a crowded intracellular environment in vitro dramatically improves RT-PCR performance Biochem Biophys Res Commun 2007 363 171 177 10.1016/j.bbrc.2007.08.156 17854768

[B15] Rozen S Skaletsky HJ Krawetz S, Misener S Primer3 on the WWW for general users and for biologist programmers Bioinformatics Methods and Protocols: Methods in Molecular Biology 2000 Totowa: Humana Press 365 386 10.1385/1-59259-192-2:365 10547847

[B16] Orly J Rei Z Greenberg NM Richards JS Tyrosine kinase inhibitor AG18 arrests follicle-stimulating hormone- induced granulosa cell differentiation: use of reverse transcriptase- polymerase chain reaction assay for multiple messenger ribonucleic acids Endocrinology 1994 134 2336 2346 7514996 10.1210/endo.134.6.7514996

[B17] Snedecor G Cochrane W Statistical Methods 1989 Ames: Iowa State University Press

[B18] Breier BH Vickers MH Ikenasio BA Chan KY Wong WP Fetal programming of appetite and obesity Mol Cell Endocrinol 2001 185 73 79 10.1016/S0303-7207(01)00634-7 11738796

[B19] Vickers MH Krechowec SO Breier BH Is later obesity programmed in utero? Curr Drug Targets 2007 8 923 934 10.2174/138945007781386857 17691929

[B20] Dahlgren J Nilsson C Jennische E Ho HP Eriksson E Niklasson A Bjorntorp P Albertsson Wikland K Holmang A Prenatal cytokine exposure results in obesity and gender-specific programming Am J Physiol Endocrinol Metab 2001 281 E326 E334 11440909 10.1152/ajpendo.2001.281.2.E326

[B21] Vickers MH Breier BH Cutfield WS Hofman PL Gluckman PD Fetal origins of hyperphagia, obesity, and hypertension and postnatal amplification by hypercaloric nutrition Am J Physiol Endocrinol Metab 2000 279 E83 E87 10893326 10.1152/ajpendo.2000.279.1.E83

[B22] Lukaszewski MA Mayeur S Fajardy I Delahaye F Dutriez-Casteloot I Montel V Dickes-Coopman A Laborie C Lesage J Vieau D Breton C Maternal prenatal undernutrition programs adipose tissue gene expression in adult male rat offspring under high-fat diet Am J Physiol Endocrinol Metab 2011 301 E548 E559 10.1152/ajpendo.00011.2011 21712534

[B23] Mark PJ Augustus S Lewis JL Hewitt DP Waddell BJ Changes in the placental glucocorticoid barrier during rat pregnancy: impact on placental corticosterone levels and regulation by progesterone Biol Reprod 2009 80 1209 1215 10.1095/biolreprod.108.073650 19208548 PMC2849810

[B24] Fan C Zirpoli H Qi K n-3 fatty acids modulate adipose tissue inflammation and oxidative stress Curr Opin Clin Nutr Metab Care 2013 16 124 132 10.1097/MCO.0b013e32835c02c8 23222801

[B25] Boullu-Ciocca S Paulmyer-Lacroix O Fina F Ouafik L Alessi MC Oliver C Grino M Expression of the mRNAs coding for the glucocorticoid receptor isoforms in obesity Obes Res 2003 11 925 929 10.1038/oby.2003.127 12917495

[B26] Paterson JM Morton NM Fievet C Kenyon CJ Holmes MC Staels B Seckl JR Mullins JJ Metabolic syndrome without obesity: Hepatic overexpression of 11beta-hydroxysteroid dehydrogenase type 1 in transgenic mice Proc Natl Acad Sci U S A 2004 101 7088 7093 10.1073/pnas.0305524101 15118095 PMC406470

[B27] Seckl JR Walker BR Minireview: 11beta-hydroxysteroid dehydrogenase type 1- a tissue-specific amplifier of glucocorticoid action Endocrinology 2001 142 1371 1376 11250914 10.1210/endo.142.4.8114

[B28] Burton PJ Waddell BJ Dual function of 11beta-hydroxysteroid dehydrogenase in placenta: modulating placental glucocorticoid passage and local steroid action Biol Reprod 1999 60 234 240 10.1095/biolreprod60.2.234 9915986

[B29] Waddell B Bollen M Wyrwoll C Mori T Mark P Developmental programming of adult adrenal structure and steroidogenesis: effects of fetal glucocorticoid excess and postnatal dietary omega-3 fatty acids J Endocrinol 2010 205 2 171 178 10.1677/JOE-09-0459 20144979

[B30] Gnanalingham MG Mostyn A Symonds ME Stephenson T Ontogeny and nutritional programming of adiposity in sheep: potential role of glucocorticoid action and uncoupling protein-2 Am J Physiol Regul Integr Comp Physiol 2005 289 R1407 R1415 10.1152/ajpregu.00375.2005 16002557

[B31] Wyrwoll CS Mark PJ Waddell BJ Developmental programming of renal glucocorticoid sensitivity and the renin-angiotensin system Hypertension 2007 50 579 584 10.1161/HYPERTENSIONAHA.107.091603 17664394

[B32] Nyirenda MJ Lindsay RS Kenyon CJ Burchell A Seckl JR Glucocorticoid exposure in late gestation permanently programs rat hepatic phosphoenolpyruvate carboxykinase and glucocorticoid receptor expression and causes glucose intolerance in adult offspring J Clin Invest 1998 101 2174 2181 10.1172/JCI1567 9593773 PMC508805

[B33] Mark PJ Lewis JL Jones ML Keelan JA Waddell BJ The inflammatory state of the rat placenta increases in late gestation and is further enhanced by glucocorticoids in the labyrinth zone Placenta 2013 34 7 559 566 10.1016/j.placenta.2013.04.006 23639575

[B34] Sarr O Yang K Regnault TR In utero programming of later adiposity: the role of fetal growth restriction J Pregnancy 2012 2012 134758 23251802 10.1155/2012/134758 PMC3518064

[B35] Jacobi D Stanya KJ Lee CH Adipose tissue signaling by nuclear receptors in metabolic complications of obesity Adipocyte 2012 1 4 12 10.4161/adip.19036 22916336 PMC3423221

[B36] Staels B Koenig W Habib A Merval R Lebret M Torra IP Delerive P Fadel A Chinetti G Fruchart JC Najib J Maclouf J Tedgui A Activation of human aortic smooth-muscle cells is inhibited by PPARalpha but not by PPARgamma activators Nature 1998 393 790 793 10.1038/31701 9655393

[B37] Blaak E Gender differences in fat metabolism Curr Opin Clin Nutr Metab Care 2001 4 499 502 10.1097/00075197-200111000-00006 11706283

[B38] Benkalfat NB Merzouk H Bouanane S Merzouk SA Bellenger J Gresti J Tessier C Narce M Altered adipose tissue metabolism in offspring of dietary obese rat dams Clin Sci (Lond) 2011 121 19 28 10.1042/CS20100534 21288203

[B39] Evans RD Williamson DH Comparison of effects of platelet-activating factor and tumour necrosis factor-alpha on lipid metabolism in adrenalectomized rats in vivo Biochim Biophys Acta 1991 1086 191 196 10.1016/0005-2760(91)90007-5 1932101

[B40] Plomgaard P Fischer CP Ibfelt T Pedersen BK van Hall G Tumor necrosis factor-alpha modulates human in vivo lipolysis J Clin Endocrinol Metab 2008 93 543 549 10.1210/jc.2007-1761 18029463

[B41] Harris WS n-3 fatty acids and serum lipoproteins: human studies Am J Clin Nutr 1997 65 1645S 1654S 9129504 10.1093/ajcn/65.5.1645S

[B42] Rustan AC Nossen JO Christiansen EN Drevon CA Eicosapentaenoic acid reduces hepatic synthesis and secretion of triacylglycerol by decreasing the activity of acyl-coenzyme A:1,2-diacylglycerol acyltransferase J Lipid Res 1988 29 1417 1426 2853717

[B43] Shirouchi B Nagao K Inoue N Ohkubo T Hibino H Yanagita T Effect of dietary omega 3 phosphatidylcholine on obesity-related disorders in obese Otsuka Long-Evans Tokushima fatty rats J Agric Food Chem 2007 55 7170 7176 10.1021/jf071225x 17661494

[B44] Wang YX Lee CH Tiep S Yu RT Ham J Kang H Evans RM Peroxisome-proliferator-activated receptor delta activates fat metabolism to prevent obesity Cell 2003 113 159 170 10.1016/S0092-8674(03)00269-1 12705865

[B45] Flachs P Rossmeisl M Bryhn M Kopecky J Cellular and molecular effects of n-3 polyunsaturated fatty acids on adipose tissue biology and metabolism Clin Sci (Lond) 2009 116 1 16 10.1042/CS20070456 19037880

[B46] Weyer C Foley JE Bogardus C Tataranni PA Pratley RE Enlarged subcutaneous abdominal adipocyte size, but not obesity itself, predicts type II diabetes independent of insulin resistance Diabetologia 2000 43 1498 1506 11151758 10.1007/s001250051560

[B47] Lonn M Mehlig K Bengtsson C Lissner L Adipocyte size predicts incidence of type 2 diabetes in women FASEB J 2009 24 1 326 331 19741173 10.1096/fj.09-133058

[B48] Jernas M Palming J Sjoholm K Jennische E Svensson PA Gabrielsson BG Levin M Sjogren A Rudemo M Lystig TC Carlsson B Carlsson LM Lönn M Separation of human adipocytes by size: hypertrophic fat cells display distinct gene expression FASEB J 2006 20 1540 1542 10.1096/fj.05-5678fje 16754744

[B49] Winkler G Kiss S Keszthelyi L Sapi Z Ory I Salamon F Kovacs M Vargha P Szekeres O Speer G Karádi I Sikter M Kaszás E Dworak O Gerö G Cseh K Expression of tumor necrosis factor (TNF)-alpha protein in the subcutaneous and visceral adipose tissue in correlation with adipocyte cell volume, serum TNF-alpha, soluble serum TNF-receptor-2 concentrations and C-peptide level Eur J Endocrinol 2003 149 129 135 10.1530/eje.0.1490129 12887290

